# Characteristics of the gut microbiota in bipolar depressive disorder patients with distinct weight

**DOI:** 10.1111/cns.14078

**Published:** 2023-01-05

**Authors:** Peifen Zhang, Danhua Zhang, Jianbo Lai, Yaoyang Fu, Lingling Wu, Huimin Huang, Yanmeng Pan, Jiajun Jiang, Caixi Xi, Ziyuan Che, Xueqin Song, Shaohua Hu

**Affiliations:** ^1^ Department of Psychiatry, The First Affiliated Hospital Zhejiang University School of Medicine Hangzhou China; ^2^ The First Affiliated Hospital Zhengzhou University Zhengzhou China; ^3^ The Key Laboratory of Mental Disorder's Management in Zhejiang Province Hangzhou China; ^4^ Brain Research Institute of Zhejiang University Hangzhou China; ^5^ MOE Frontier Science Center for Brain Science & Brain‐Machine Integration Zhejiang University; ^6^ Wenzhou Medical University Wenzhou China; ^7^ College of Agriculture & Biotechnology Zhejiang University Hangzhou China

**Keywords:** 16 S rRNA, bipolar disorder, gut microbiota, metabolism, weight

## Abstract

**Background:**

Preliminary studies have indicated metabolic dysfunction and gut dysbiosis in patients with bipolar disorder (BD). In this study, we aimed to clarify the impact of the gut microbial composition and function on metabolic dysfunction in BD patients with an acute depressive episode.

**Methods:**

Fresh fecal samples were provided from 58 patients with BD depression, including 29 with normal weight (NW) and 29 with overweight/obesity (OW), and 31 healthy controls (HCs). The hypervariable region of 16 S rRNA gene (V3‐V4) sequencing was performed using *IonS5TMXL* platform to evaluate the bacterial communities. Differences of microbial community and correlation to clinical parameters across different groups were analyzed.

**Results:**

Compared to NW and HCs, the OW group showed a decreased tendency in alpha diversity index. Beta diversity was markedly different among these groups (PERMANOVA: *R* ^*2*^ = 0.034, *p* = 0.01) and was higher in patients versus HCs. A total number of 24 taxa displayed significantly different abundance among OW, NW, and HCs. At the family level, the abundance of three taxa was remarkably increased in NW, one in OW, and one in HCs. At the genus level, five taxa were enriched in OW, eight in NW, and two in HCs. The relative abundance of the genera *Megamonas* was positively associated with BMI, while *Eggerthella* was negatively correlated with BMI. Functional prediction analysis revealed the metabolism of cofactors and vitamins and amino acid were highly enriched in OW compared to HCs. In addition, microbial functions involved in “lipid metabolism” were depleted while the “fructose and mannose metabolism” was enriched in OW compared to NW group.

**Conclusions:**

Specific bacterial taxa involved in pathways regulating the lipid, energy, and amino acid metabolisms may underlie the weight concerns in depressed BD patients. Potential targeting gut microbial therapy is provided for overweight/obesity patients with BD, which still need further studies in the future.

## Graphical abstract

Preliminary studies have indicated metabolic dysfunction and gut dysbiosis in patients with bipolar disorder (BD). In this study, we aimed to clarify the impact of the gut microbial composition and function on metabolic dysfunction in BD patients with an acute depressive episode. Fresh fecal samples were provided from 58 patients with BD depression, including 29 with normal weight (18.5 kg/m^2^ ≤ BMI ≤ 23.9 kg/m^2^) and 29 with overweight/obesity (BMI ≥ 24 kg/m^2^), and 31 healthy controls (normal weight: 18.5 kg/m^2^ ≤ BMI ≤ 23.9 kg/m^2^). The hypervariable region of 16S rRNA gene (V3‐V4) sequencing was performed using *IonS5TMXL* platform to evaluate the differences of microbial community and correlations with clinical parameters. Potential targeting gut microbial therapy is provided for metabolic dysfunction in patients with BD.

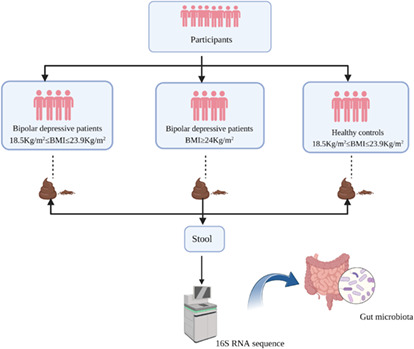

## 1 INTRODUCTION

Bipolar disorder (BD) is a severe, chronic, and recurrent mental disorder that concerns more than 1% of the worldwide population.^1^ BD typically manifests as alternative episodes of (hypo) mania and depression and intermittent remission.^2^ Onset of individuals with BD usually occurs around the adolescence, and in recent years, BD has emerged as one of the leading causes of disability and mortality worldwide, which is closely related with high comorbidity with cardiovascular and other metabolic diseases.^3^, ^4^

Overweight and obesity are well‐known risk factors for cardiovascular diseases. Individuals with BD are at a higher risk for developing obesity or overweight, even in drug‐naïve patients.^5^ Compared to the general population, about 70% of individuals with BD were troubled with obesity or overweight.^6^ Compared to normal‐weight patients, mood swings in obese BD patients were more frequent, with a shortened interval of euthymia and poorer response to the medications.^7^, ^8^ The body mass index (BMI) of BD patients was positively correlated with suicide attempts.^7^ Even in the euthymic phase, the negative relationship between BMI and the attention and processing speed was observed.^9^ These evidences indicated a high prevalence of weight problems in BD patients, which may worsen the illness severity, cognitive functions, and prognosis of patients. However, the mechanisms underlying metabolic disturbances in BD patients are still insufficiently elucidated.

Recently, mounting studies have showed that the commensal gut microbiota played an essential role in modulating human health and diseases. The putative role of the microbial dysbiosis in mediating the pathogenesis of obesity has come to the surface. Changes in the fecal richness of *Firmicutes* and *Bacteroidetes* were observed in obese subjects.^10^, ^11^ Microbial butyrate producers were more prominent in patients with higher BMI.^12^ After receiving obesity‐associated microbiota via transplantation, the weight of lean mice was recovered.^13^ Prebiotic inulin supplementations can reduce the BMI, suppress the adiposity, and mediate hepatic steatosis.^14^ In addition, gut microbiota and its metabolites could directly or indirectly influence the host's feeding behavior via stimulating the neuroendocrine release.^15^, ^16^ Existing evidence also showed that the dysbiosis of gut microbiota occurred in BD patients and some microbial biomarkers may be useful for BD diagnosis and treatment predication.^17^, ^18^, ^19^ However, the alterations of gut microbiota in BD patients with distinct weight have not been systematically studied up till now.

Therefore, we proposed the hypothesis that gut microbiota may be involved in the weight changes in BD patients. The aim of the present study is to thus compare the difference of gut microbiota, decipher its relationship with clinical profiles, and predict the functional pathways of microbial genes in BD patients with differed weight.

## 2 METHODS

### 2.1 Study subjects

BD inpatients/outpatients with current depressive episodes were recruited by a convenience sampling method from the Department of Psychiatry, the First Affiliated Hospital, Zhejiang University School of Medicine from 2017 to 2020. According to the Diagnostic and Statistical Manual of Mental Disorders, 4th Edition (*DSM‐IV*), the diagnosis of BD was made based on comprehensive clinical interviews by two senior psychiatrists using the Mini International Neuropsychiatric Interview (M.I.N.I.) for *DSM‐IV*. The inclusion criteria were as follows: (1) Scoring ≥14 on the Hamilton Depression Rating Scale (HAMD‐24) was set as the primary enrollment criteria and (2) drug‐naive or free from psychotropics for at least 3 months. Healthy volunteers without a family or personal history of psychiatric disorders were recruited through advertisement. Exclusion criteria for all subjects included serious physical comorbidities (e.g., heart failure, liver cirrhosis, hematological diseases, and malignancy), alcohol or substance abuse, acute or chronic infection, autoimmune diseases, pregnancy or breastfeeding women, and taking antibiotic or probiotics/prebiotics supplement within 4 weeks prior to sampling.

### 2.2 Clinical characteristics

Demographic and clinical profiles, including age, gender, duration of illness, family history, and educational level, were collected through face‐to‐face interviews. The Montgomery‐Åsberg Depression Rating Scale (MADRS) and HAMD‐24 were used to assess the severity of depression. The Young Manic Rating Scale (YMRS) was used to evaluate the severity of mania. The Hamilton Anxiety Rating Scale (HAMA) was used to estimate the severity of anxiety. The weight and height of all subjects were measured to calculate the BMI value as dividing weight (kg) by height (m) squared. In our study, the overweight/obese was defined as a BMI value not <24 kg/m^2^ and normal weight as BMI from 18.5 to 23.9 kg/m^2^ ^20^

### 2.3 Fecal samples

Fecal samples from each individual were collected using fecal containers. The collected samples were stored in the refrigerator under −80°C prior to processing.

### 2.4 DNA Extraction and polymerase chain reaction (PCR) amplification

The microbial DNA from the frozen fecal samples was extracted with the Magnetic Soil and Stool DNA Kit (TIANGEN BIOTECH) according to the manufacturer's instructions. After evaluating the concentration and purity, the reactions of PCR were conducted with Bio‐Rad T100 Gradient PCR Instrument. The V3‐V4 region of the16S rRNA was amplified with 341F (5′‐barcode‐CCTAYGGGRBGCASCAG‐3′) and 806R (5′‐GGACTACNNGGGTATCTAAT −3′) primers set. All PCRs were carried out with Phusion Master Mix (New England BioLabs Inc). The PCR products were mixed and purified with GeneJET PCR Purification Kit (Thermo Scientific) according to the protocol provided by the manufacturer.^21^

### 2.5 16 S rRNA Gene sequence analysis

The amplicon library was prepared with TruSeq® DNA PCR‐Free Library Preparation Kit (Illumina, USA) and quantified with Qubit (Thermo Scientific). Finally, the library was sequenced on an IonS5TMXL platform (Tianjin, China). 396–415 bp single‐end reads for each sample were generated. Totally, samples resulted in 7,008,024 clean reads and 78,741 per sample in average, and 1214 Operation Taxonomic Units (OTUs) were clustered through 97% identity and annotated via the GreenGenes database (version 13_8).

Alpha diversity indices (Shannon, Simpson, chao1, and ACE diversity) were calculated with the Wilcoxon rank sum test (R packages, v.4.1.0) to evaluate differences among these samples. The principal coordinate analysis (PCoA) was conducted using the “vegan package,” and the permutational multivariate analysis of variance (PERMANOVA) was calculated to estimate variability of the beta diversity among groups. Differences in compositions of the microbial taxa among these three groups were estimated through the linear discriminant analysis (LDA) effect size (LEfSe) method (http://huttenhower.sph.harvard.edu/galaxy). An alpha significance level of 0.05 and the LDA (log10) scores cutoff of 2 were set threshold to identify microbial biomarkers. The functional profiles of the bacterial gene were predicted through the phylogenetic investigation of communities by reconstruction of unobserved states (PICRUSt).

### 2.6 Statistical analysis

The IBM SPSS Statistics (Version 21) and R software (version.4.1.0) were used for the statistical analysis. Comparisons for normal continuous variables among these three groups were performed with one‐way analysis of variance (ANOVA). Chi‐square test was carried out for categorical data. Difference of the non‐normal continuous variables was analyzed by using the Kruskal–Wallis test. Correlations between clinical variables and specific bacterial taxa in patients were performed using Spearman's correlation analysis and visualized by “pheatmap package.” The ggplot2 package was used to produce plots or violin visualizations. The standard of the statistical significance was set as *p* values <0.05.

## 3 RESULTS

### 3.1 Clinical characteristics of the recruited subjects

In total, 89 subjects were recruited in this study, including 58 type II BD patients with a current depressive episode and 31 healthy controls (HCs). Subsequently, BD patients were further divided into two groups: the normal body weight (NW) and the BD patients with overweight/obese (OW) according to the aforementioned BMI criterion. Gender, age, and educational level are well matched among these three groups. The general clinical characteristics of OW, NW, and HCs groups are summarized and presented in Table 1. When compared to the HCs group, higher HAMA, HAMD, MADRS, and YMRS scores were presented in OW and NW groups (*p*<0.05). In OW group, the weight and BMI values were significantly the highest (*p*<0.05).

**TABLE 1:** Demographic characteristics and clinical data in all recruited subjects.

	BD (*n* = 58)		HCs (*n* = 31)	*F/K‐W/χ* ^*2*^	*p*
	Normal weight (*n* = 29)	Overweight/obese (*n* = 29)	Normal weight (*n* = 31)		
Age (years)	22.9 ± 6.07	26.26 ± 10.85	21.29 ± 1.92	0.803^b^	0.669
Gender, *n* (%)					
Male	16(55)	18(62)	13(42)	2.534^c^	0.282
Female	13(45)	11(38)	18(58)		
Height (cm)	167.28 ± 7.85	169.76 ± 8.71	167.77 ± 9.06	1.027^a^	0.363
Weight (kg)	55.95 ± 6.09	77.40 ± 11.21	57.97 ± 7.97	54.52^a^	<0.001
BMI (kg/m^2^)	19.95 ± 1.11	26.79 ± 2.81	20.77 ± 1.67	59.30^b^	<0.001
Education (years)	14.48 ± 3.23	14.23 ± 3.56	15.61 ± 1.56	1.97^a^	0.146
Duration of illness (years)	4.40 ± 3.94	6.44 ± 7.34	/	−0.319^d^	0.749
Family history					
Yes	2	3	/	0.220^c^	0.50
No	25	24	/		
Null	2	2	/		
HAMA	24.28 ± 6.93	23.17 ± 8.51	2.32 ± 2.30	60.47^b^	<0.001
HAMD	33.38 ± 6.73	32.45 ± 9.34	2.32 ± 2.76	60.44^b^	<0.001
YMRS	7.21 ± 7.96	4.03 ± 6.92	0.87 ± 1.23	26.08^b^	<0.001
MADRS	29.10 ± 6.39	26.72 ± 9.38	1.48 ± 2.11	58.08^b^	<0.001

*Note*: F (a): One Way ANOVA. K‐W (b): Kruskal‐Wallis. *χ* ^2^ (c): Chi‐square test. (d): Wilcoxon rank sum test. *p*: *p*‐value. Data are shown as the mean ± standard deviation.

Abbreviations: BD, Bipolar disorder; BMI, Body Mass Index; HAMA, Hamilton Anxiety Rating Scale; HAMD, Hamilton Depression Rating Scale; HCs, Healthy controls; MADRS, Montgomery‐Åsberg Depression Rating Scale; YMRS, Young Mania Rating Scale.

### 3.2 Gut microbial diversity

**FIGURE 1:**
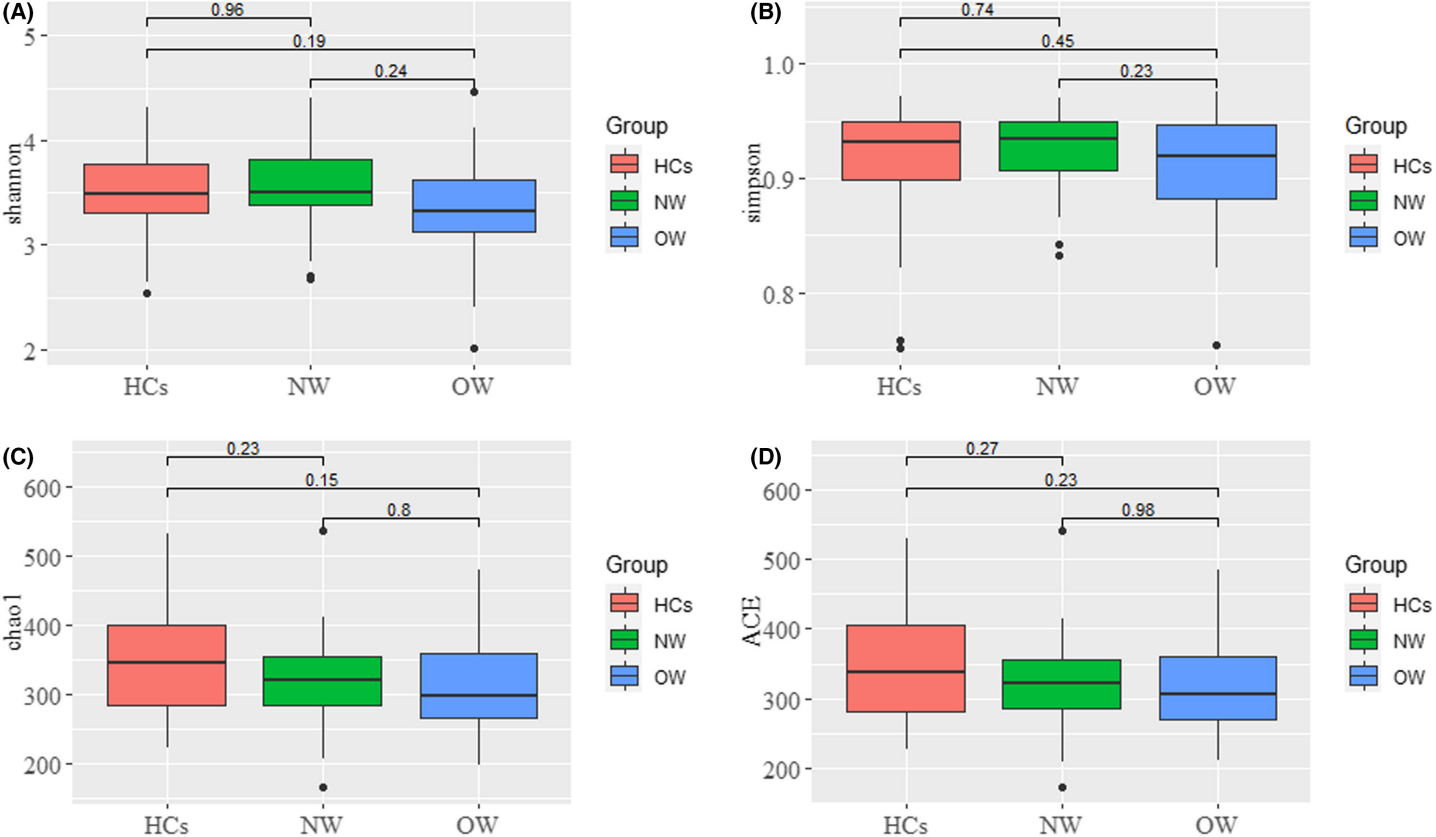
Microbial alpha diversity in the OW, NW, and HCs groups. (A–D) Box plots present divergences in the microbial diversity indices among OW, NW, and HCs groups according to the Shannon, Simpson, chao 1, and ACE, respectively, based on the Operation taxonomic unit level. OW: Patients with overweight/obesity; NW: Patients with normal weight; HCs: Healthy controls.

As shown in Figure 1, the alpha diversity of the OW group was the lowest (Shannon, Simpson, chao 1 and ACE), albeit no statistical difference was observed when compared to the NW or HCs groups.

**FIGURE 2:**
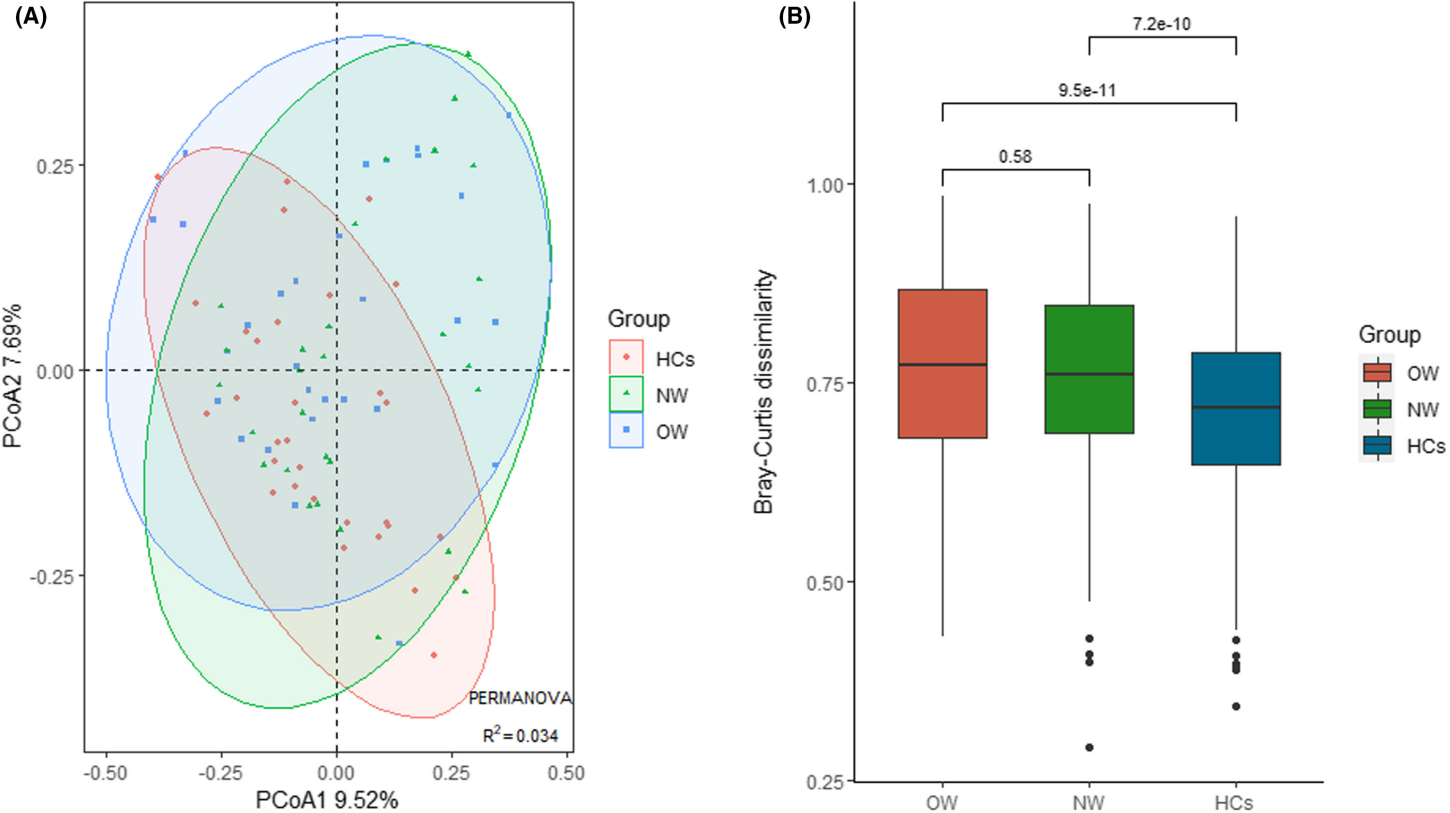
Beta diversity based on the Bray‐Curtis distances of the fecal microbiota in the OW, NW, and HCs groups. (A): The plot presented the first two principal coordinates (PC1, PC2) for PCoA. (B): Difference of the beta diversity for OW, NW, and HCs groups. PCOA: Principal coordinates analysis; OW: Patients with overweight/obesity; NW: Patients with normal weight; HCs: Healthy controls.

Based on the Bray–Curtis distance at the OUTs level, the analysis of PCoA presented a marked difference in the OW and NW groups when compared to the HCs group in the first two principal coordinates (PC1, PC2), confirmed by the PERMANOVA test (R^2^ = 0.034, *p* = 0.01) (Figure 2A). This result indicated the intergroup variability of the bacterial community had a clear separation among these groups. Additionally, compared to HCs, the beta diversity was higher in both OW and NW groups (Figure 2B).

### 3.3 Differential taxonomic compositions of gut microbiota in OW, NW, and HCs groups

**FIGURE 3:**
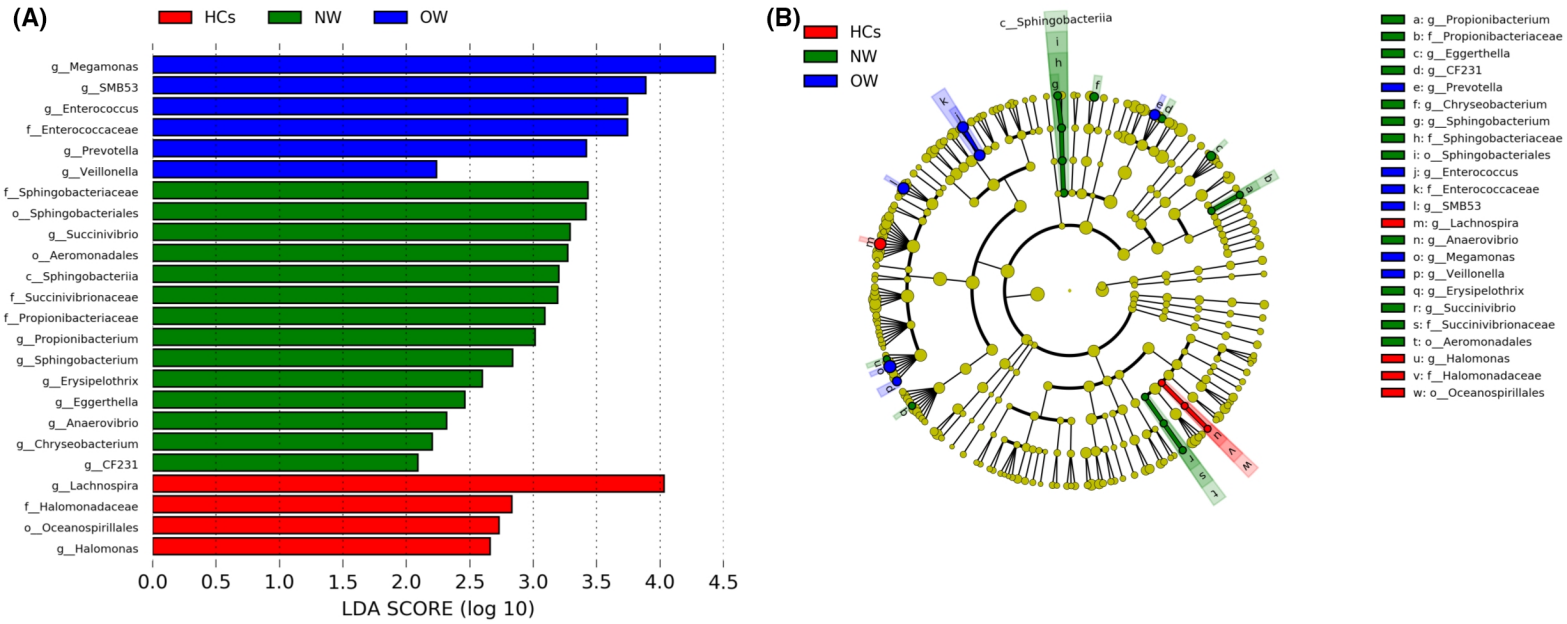
Differential gut microbiota taxa were analyzed through the LEfSe analysis. (A): LDA scores plots showed that the compositions of the microbial taxa were significantly differential among OW (blue), NW (green) and HCs (red) groups. (B): The cladogram of different microbial taxa in OW, NW, and HCs groups. OW: Patients with overweight/obesity; NW: Patients with normal weight; HCs: Healthy controls.

The LEfSe analysis showed a total of 24 bacterial taxa changed in the OW, NW, and HCs groups (the LDA score >2, *p* < 0.05). Among them, 4 bacterial taxa were enriched in HCs, 6 in OW, and 14 in NW. At the family level, the abundance of *Halomonadaceae* was enriched in HCs, *Enterococcaceae* were dominated in OW, while *Propionibacteriaceae* and *Sphingobacteriaceae* were more prevalent in NW. Multiple genera, including *Megamonas*, *SMB53*, *Enterococcus*, *Veillonella*, and *Prevotella*, were enriched in OW. The genera *Succinivibrio*, *Propionibacterium*, *Sphingobacterium*, *Erysipelothrix*, *Eggerthella*, *Anaerovibrio*, *Chryseobacterium*, and *CF231* were dominated in NW. The abundance of the genera *Lachnospira* and *Halomonas* was higher in HCs (Figure 3A,B).

### 3.4 Associations between gut microbiota and clinical parameters

**FIGURE 4:**
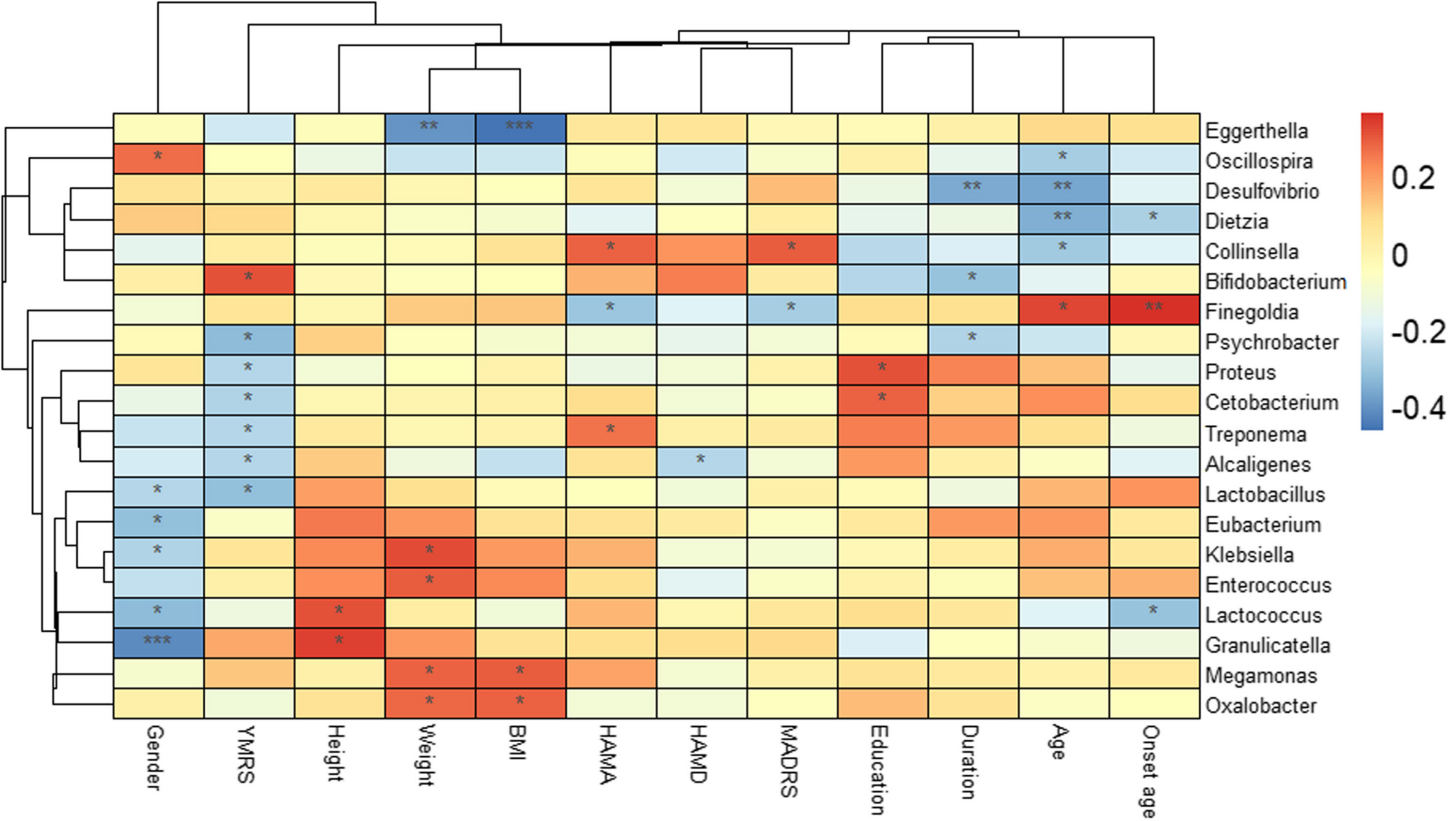
Correlations of the fecal microbiota at the genus level with clinical characteristics were presented in the heatmap. * *p* < 0.05, **p < 0.01, ***p < 0.001.

Correlations between the identified bacterial genera and the BD clinical parameters were evaluated. Results showed that there was an obviously negative relationship between BMI and the abundance of *Eggerthella*, while *Megamonas* and *Oxalobacter* were positively correlated with BMI. In addition, a negative correlation was observed between the severity of depression and the abundance of genera *Alcaligenes* (Figure 4).

### 3.5 Functional Prediction Analysis

**FIGURE 5:**
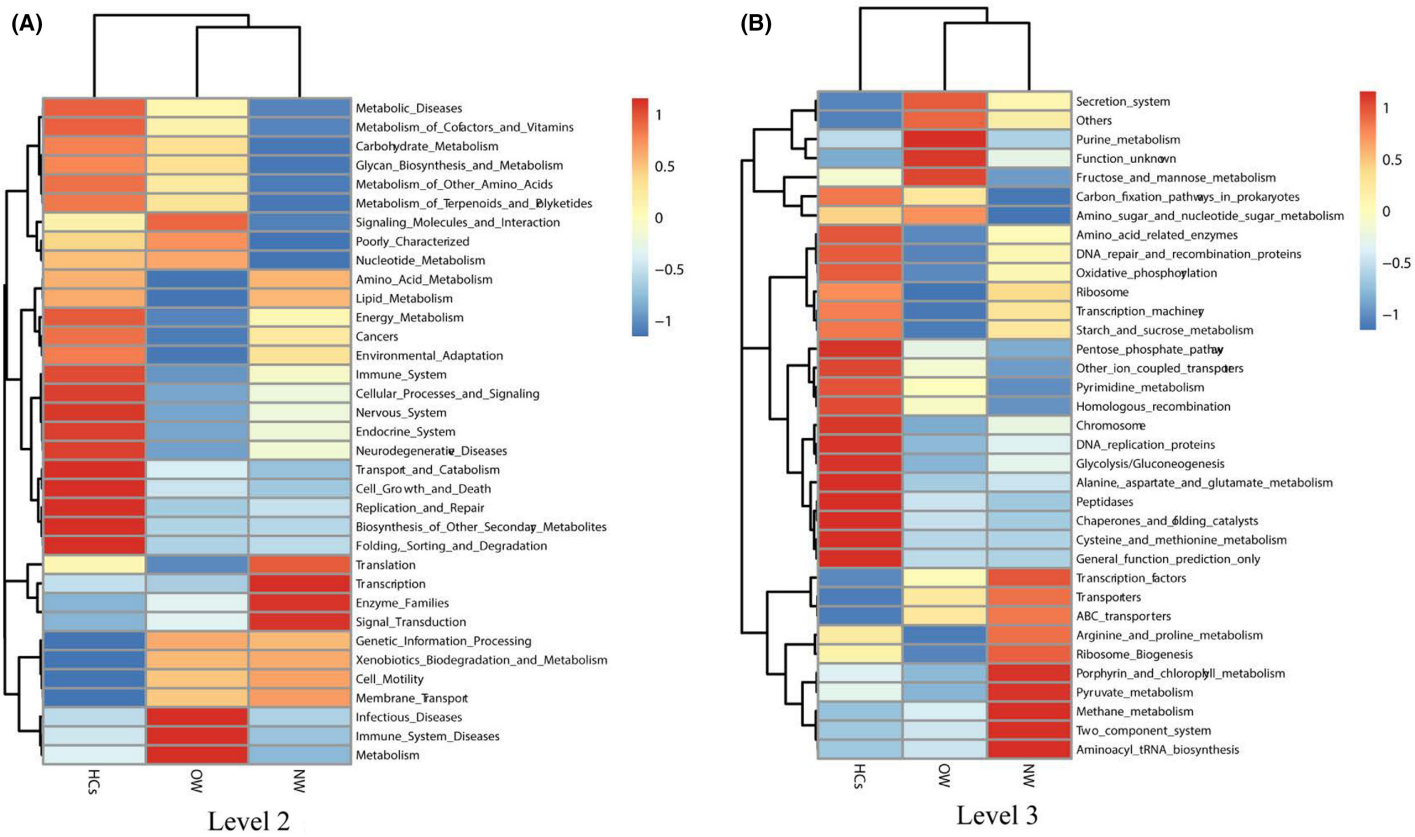
The cluster heatmap showed the predicted function pathways of the microbial gene at the level 2 and 3 KEGG database. OW: Patients with overweight/obesity; NW: Patients with normal weight; HCs: Healthy controls.

Based on the OTUs' reference sequence, the PICRUSt was used to predict the functional capacities of the bacterial genes. The heatmap presented the differential genes cluster features at levels 2 and 3 of the Kyoto Encyclopedia of Genes and Genomes (KEGG) pathways, respectively (Figure 5A,B).

**FIGURE 6:**
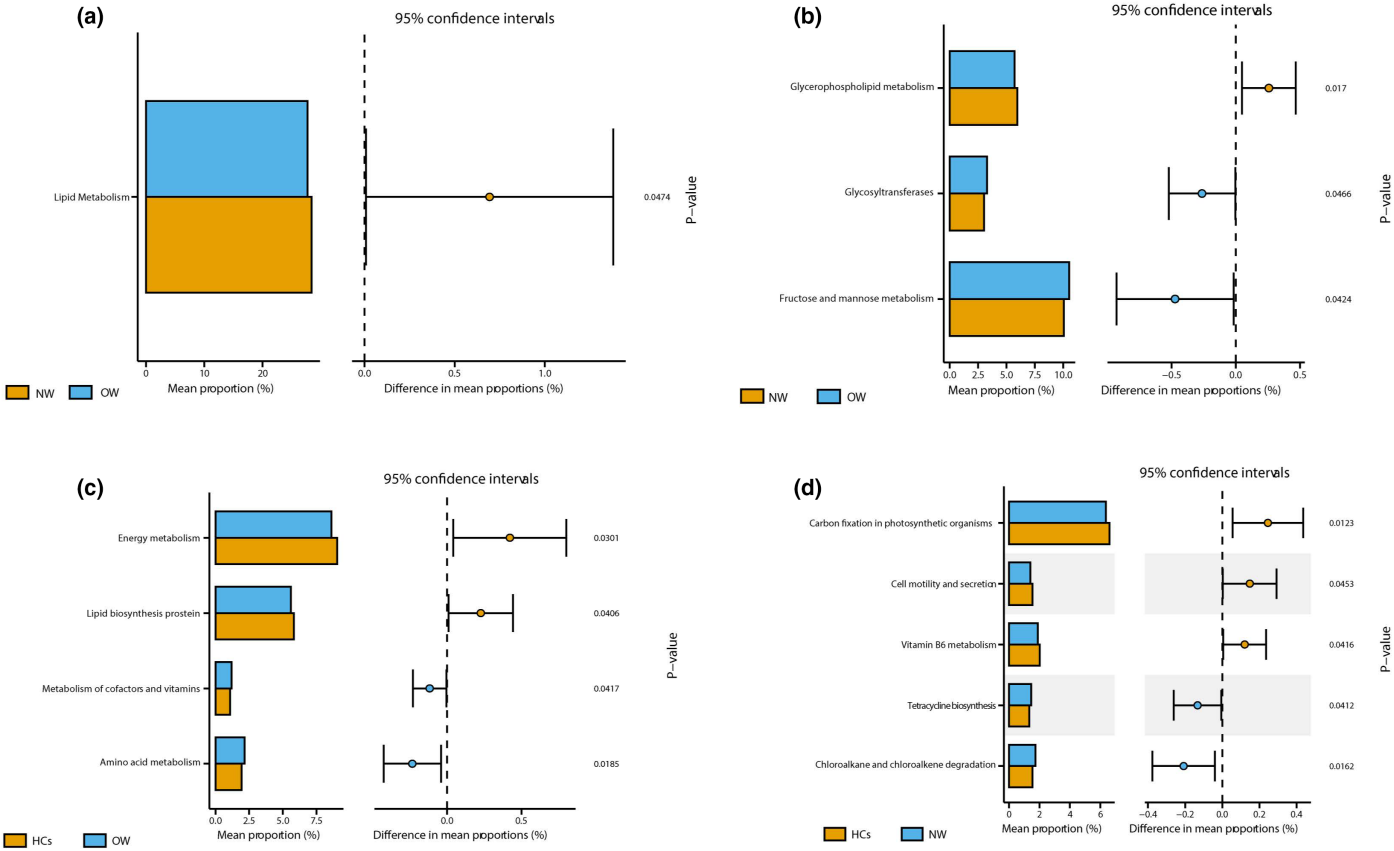
Predicted function of the microbial genes in the OW, NW, and HCs groups. (A): Significant different KEGG pathways at level 2 for the gut microbiota in OW and NW using *t*‐test. (B–D): Significant different KEGG pathways at level 3 for the gut microbiota among OW, NW and HCs groups. OW: Patients with overweight/obesity; NW: Patients with normal weight; HCs: Healthy controls; KEGG, Kyoto Encyclopedia of Genes and Genomes.

In the level 2 KEGG pathways, the function of microbial gene that involved in the lipid metabolism was significantly enriched in NW group (Figure 6A). In the level 3, there were totally12 KEGG pathways generated among OW, NW, and HCs groups. In details, compared to NW group, the glyceropholipid metabolism was reduced while the pathway of fructose and mannose metabolism and the glycosyltransferases were increased in the OW group (Figure 6B). Compared to the HCs group, the function of microbial gene that involved in the lipid biosynthesis proteins and energy metabolism was significantly reduced in the OW group while the amino acid metabolism and metabolism of cofactors and vitamins were highly enriched in the OW group (Figure 6C). For the NW group, the carbon fixation in photosynthetic organism, cell motility and secretion and Vitamin B6 metabolism was decreased, while the tetracycline biosynthesis, chloroalkane and chloroalkene degradation were enriched (Figure 6D).

## 4 DISCUSSION

This study provided evidence that depressed BD patients with distinct weight displayed different gut microbial communities. Associations between the identified gut microbiota and clinical characteristics as well as the microbial functional pathways indicated that the dysbiosis of the gut microbiota was possibly involved in the pathogenesis of BD with distinct weight through mediating the lipid, amino acid and energy metabolisms.

Our results showed that the microbial diversity was different among these groups. Microbial richness and evenness, as evaluated by the alpha diversity indexes, had a diminished trend in the OW group, albeit this difference had no significant statistical significance. This fingding was consistent with the study of Martínez‐Cuesta et al.^22^ However, beta diversity in patients was increased in our study. When fed with a high‐fat diet for 12 weeks, the beta diversity of gut microbiota in mice was increased in parallel with the weight.^23^ These results indicated that an increased beta diversity may be linked to the weight gain. However, data from literatures regarding the microbial diversity in obese individuals are inconsistent due to various factors, such as age, dietary patterns, physical fitness, and geographical differences.

In this study, the taxonomic signatures of microbiota in BD patients with distinct weight were distinguished from those of HCs. In the level of genera, *Megamonas*, *SMB53*, *Enterococcus*, and *Veillonella*, which belong to the phylum *Firmicutes*, were the dominant species in the OW group. While *Sphingobacterium* and *CF231* belonging to phylum *Bacteroidetes* were higher in the NW and HCs groups, respectively. This finding is in line with the Palmas et al.'s^24^ study and provides further clues that the gut microbial dysbiosis existed in overweight/obese patients with BD. Interestingly, the amount of the phyla *Firmicutes* and *Bacteroidetes* accounts for over 90% of the distal gut microbiota.^25^ An increased *Firmicutes* to *Bacteroidetes* (F/B) phylum ratio was related with the obese feature.^26^ The phyla *Firmicutes* produce the short‐chain fatty acids (SCFAs) via metabolizing dietary fibers, which can accelerate energy accumulation and lipogenesis following high‐carbohydrate diets.^27^, ^28^ In addition, the phyla *Firmicutes* encoded less carbohydrate‐degrading enzymes than *Bacteroidetes*.^29^ These evidences theoretically indicate that the phyla *Firmicutes* aggravate obesity while *Bacteroidetes* attenuate overweight/obesity. However, a few studies found no statistically significant or even opposite alterations of the phyla *Firmicutes* and *Bacteroidetes* in distinct weight groups.^30^ These discordant results may be attributed to the different sized sample, clinical features, and sequencing methods across different studies.

A close correlation between identified microbiota and clinical features in this study further indicated the potential effects of the microbiota on the weight. The positive correlation between the genera *Megamonas* and BMI was in line with the Ma et al.,^31^ while the negative correlation between the *Eggerthella* and BMI was in line with Sato et al.^32^ *Megamonas* is able to ferment glucose into acetic and propionic acid, thus serving as a substrate for lipogenesis and cholesterol formation and provide energy for the host.^33^ *Megamonas* spp. also carries bacterial α‐amylase, which may cause dyslipidemia through the acetyl‐CoA synthesis.^34^ Strains of *Eggerthella* are capable of metabolizing bile acids, which act as signaling molecules to regulate the lipid metabolism.^35^, ^36^, ^37^ Therefore, we assumed that these two identified genera may be involved in the disrupted lipid metabolism in depressed BD patients.

Lastly, we utilized the PICRUSt analysis to preliminarily explore the difference about microbial metabolic pathways among these samples. Herein, our results showed that microbial functions involved in “lipid metabolism” were depleted while the “fructose and mannose metabolism” was enriched in OW compared to NW group. Furthermore, the microbial genes associated with “Amino acid metabolism” and “Metabolism of cofactors and vitamins” were enriched in OW patients rather than healthy controls, all of which were consistent with the previous studies.^38^, ^39^ Changes in microbial compositions directly affect the production of microbe‐derived metabolites, including amino acids, lipids and their byproducts, and some of them can activate the ligands of the G protein‐coupled receptor, accelerate the production of peptide YY, inhibit gut motility and energy extraction from food and play crucial roles in metabolic disorders.^40^, ^41^ Furthermore, specific bacteria can not only inhibit the lipid synthesis but also increase the amino acid level via enhancing the expression of transcriptional factors.^42^ Therefore, we hypothesized that the reduced lipid and increased amino acid metabolism in our study may partly be attributed to the above clues. In addition, previous studies regarded elevations in some amino acid as risk factors for obesity.^43^, ^44^ Cofactors and vitamins could provide substance for many physiological processes, including amino acid metabolism.^45^ The abundance of pathway related to amino acid and vitamins was both increased in our study and these data indicated that gut microbiota of OW patients may have an enhanced capacity for amino acid metabolism. Certainly, the mechanism of the alterations in predicted metabolic pathways caused by microbiota in BD patients is still in its infancy and needed to be further explored.

This study has some inherent limitations. First, the sample size may be relatively small and the difference of the OTUs among these three group was weak according to the values of PERMANOVA test. Therefore, further verifications on larger sample sizes are needed. This work is a cross‐sectional study and cannot yield any causality between the gut dysbiosis and obesity. Although the recruited participants lived in the same region, other factors such as diet patterns and physical activities can also confound the final findings. Therefore, longitudinal studies with well‐designed control on mixed factors are required in the future.

## 5 CONCLUSION

Overall, this study provides support for the dysbiosis of gut microbe and its relationship with the weight changes in depressed BD patients. These findings contribute to our knowledge of the microbial roles in the pathogenesis of overweight/obesity in BD and may open new opportunities for the adjuvant therapy aiming at gut microbiota in clinicals to prevent from obesity in future. Honestly, further studies are required to obtain cause‐effect mechanism between the microbiota and overweight/obesity in BD patients.

## AUTHOR CONTRIBUTIONS

PZ was in charge of the analysis of data, interpretation of the results, and writing the manuscript. DZ and JL searched the references, interpreted the results, and revised the manuscript. YF, LW, HH, YP, CX, and ZC collected the samples. XS and SH provided the concept and revised the manuscript. All authors approved the final version of the manuscript.

## FUNDING INFORMATION

This work was supported by the National Natural Science Foundation [No. 81971271 to S.H.], the Zhejiang Provincial Key Research and Development Program [No. 2021C03107 to S.H.], the Zhejiang Provincial Natural Science Foundation [No. LQ20H090013 to J.L.], the Program from the Health and Family Planning Commission of Zhejiang Province [No. 2020KY548 to J.L.], and the Leading Talent of Scientific and Technological Innovation‐'Ten Thousand Talents Programme' of Zhejiang Province [No. 2021R52016 to S.H.].

## CONFLICT OF INTEREST

All authors declare that there are no conflicts of interest.

## Data Availability

The data that support the findings of this study are available from the corresponding author upon reasonable request. Due to the patients' privacy and ethical restrictions, the dataset is not publicly available.
